# Assessment of the Applicability of Coconut and Skim Milk Powder as a Carrier for Lactic Acid Bacteria on Their Performance During Production of *Ting*

**DOI:** 10.1155/ijfo/6198794

**Published:** 2025-08-10

**Authors:** Nontobeko Xolisiwe Zulu, Angela Parry-Hanson Kunadu, Eugenie Kayitesi, Bhekisisa Dlamini

**Affiliations:** ^1^Department of Biotechnology and Food Technology, University of Johannesburg, Johannesburg, Gauteng, South Africa; ^2^Department of Nutrition and Food Science, University of Ghana, Accra, Ghana; ^3^Department of Consumer and Food Sciences, University of Pretoria, Hatfield, Gauteng, South Africa

**Keywords:** coconut powder, fermentation, lactic acid bacteria, skim milk, *ting*

## Abstract

The demand for dried starter cultures that are specific to indigenous fermented food products is increasing. In most cases, skim milk (SM) powder is used as a cryoprotectant for microbial cell protection during the preparation of starter cultures. However, the extent of cell protection during freeze-drying and the fermentation efficacy of dried cultures are dependent on the type of microbial strain and carrier media, hence the need to investigate the protective effects of alternative carrier media. This study evaluated the fermentation potential of lactic acid bacteria (LAB) preserved in coconut (CCN) powder and SM powder during *ting* (traditional fermented sorghum) production. *Ting* was fermented with single and mixed strains of *Lactobacillus plantarum* and *Lactobacillus brevis*. The pH, total titratable acidity (TTA), functional groups (Fourier transform infrared spectroscopy [FTIR]), microbial quality, and consumer acceptability were monitored during fermentation. *Ting* prepared with LAB strains preserved in SM had a more rapid reduction (*p* > 0.05) in pH and lower final pH than *ting* prepared with LAB preserved in CCN. Mixed LAB strains showed a rapid reduction in pH of *ting* compared to single LAB strains and *ting* prepared with spontaneous fermentation. The highest TTA (3.57%) was observed with mixed LAB after 48 h on both SM and CCN *ting*. FTIR showed similar functional groups corresponding to O–H and phenolic compounds for both SM and CCN *ting*. The highest increase in LAB counts (up to 10 log CFU/mL) occurred in *ting* prepared with mixed strains of both CCN and SM, while the least increase occurred with spontaneously fermented *ting*. Enterobacteriaceae, yeasts, and molds were not detected in all the fermented samples. With consumer acceptability, CCN *ting* was the most preferred sample with the highest overall score (6.95), followed by the SM *ting* sample (5.67). In conclusion, this work indicates that the LAB strains preserved in CCN result in comparable fermentation performance to that of SM and produce *ting* that is preferred by consumers. Therefore, CCN should be considered as a carrier medium for the development of *ting* starter cultures.

## 1. Introduction

The importance of indigenous fermented food products in food security has received more attention nowadays. This is mainly because of the use of drought-resistant crops such as sorghum in the production of some fermented food products. In addition, the unique flavor profile associated with indigenous food products has attracted the interest of many scientists. The flavor profile of indigenous food products is influenced by several factors, one of which is the microbial community that drives the fermentation process [1]. Since most indigenous fermented food products rely on spontaneous fermentation, they have inconsistent food product quality and, in some cases, both spoilage and pathogenic microorganisms have been isolated from such food products [2]. There is, therefore, a need to isolate the specific microorganism responsible for the fermentation of indigenous food for the development of starter cultures. Currently, there is a paucity of starter cultures that are specific to indigenous food, and this may negatively affect the retention of unique flavor compounds in such foods.

The gold standard in the long-term preservation of dried starter cultures is the use of the freeze-drying technique. However, the quality (vitality and viability) of freeze-dried cultures is dependent on the type of cryoprotectants used during the process [3]. For most commercial starter culture preparations, skim milk (SM) is generally used as one of the cryoprotectants, that is, in the presence of other ingredients such as sugar supplements (sucrose, maltose, trehalose, dextrose, and fructose) to further protect the cell against harsh drying conditions [4, 5]. This is because SM can act as an encapsulant that encloses active cells, thus protecting against stresses such as heat [6]. Lately, the cell protective potential of nondairy carrier media from cereal sources has been explored, possibly due to consumer demands for dairy-free products and the potential of these sources to maintain cell quality and flavor properties of indigenous foods.

Cereals are beneficial producers of carbohydrates, proteins, minerals, and vitamins [7], hence their potential of being used as carrier media for starter cultures. For example, oat was reported as the best carrier medium for maintenance of *Lactobacillus curvatus* P99 as a probiotic bacteria, resulting in a fermented dairy oat beverage with high consumer acceptability [8]. Elsewhere, the use of lactic acid bacteria (LAB) prepared with maize and soluble starch as carrier media was reported to improve fermentation efficacy, resulting in stable fermentation [9, 10]. There is, therefore, a need to investigate the applicability of other nondairy carrier media in the protection of fermenting microorganisms during freeze-drying. In fact, our recent study has shown that the use of coconut (CCN) in the presence of sucrose (5%) can protect *Lactobacillus plantarum* and *Streptococcus thermophilus* cells like SM powder during freeze-drying [11]. Although this is promising, investigating the fermentative potential of such cultures on a food product is more important to confirm the protective effects of the carrier media. In this study, the fermentation efficacy of LAB strains (single or mixed) prepared with CCN as a drying carrier medium was investigated during fermentation of *ting*. The focus of the study was to assess the applicability of CCN as a dairy-free carrier of LAB when compared to *ting* prepared with LAB strains prepared with SM as carrier medium and to spontaneously fermented *ting*. *Ting* was targeted because it is produced with sorghum and primarily through the spontaneous fermentation process. In addition, *ting* is preferred in Southern Africa because of its rich nutrient profile that contains vitamins, dietary fiber, proteins, and carbohydrates and for its renowned unique tangy flavor [12].

## 2. Materials and Methods

### 2.1. Starter Cultures

The starter cultures used in this study (*L. plantarum* and *L. brevis*) were applied singly and in combination. These strains had been previously identified by sequencing of the 16S rRNA gene. Before use and during the study, the strains were characterized biochemically according to methods by [13]. The strains were prepared by freeze-drying in CCN and SM as a carrier media. The freeze-dried strains were stored for 112 days at 4°C in vacuum packs and used in a powder form. The LAB cultures were tested for cell viability prior to use [11]. The cell viability after storage was approx. 8.5 log CFU/g for *L. plantarum* and 8.10 log CFU/g for *L. brevis* in both carrier media.

### 2.2. Fermentation of Sorghum to Produce *Ting*

*Ting* was prepared by fermenting sorghum flour with single strains and mixed strains (*L. plantarum* and *L. brevis*) in both SM and CCN as a carrier media. The formulations (1–3) were prepared as indicated in Table 1 for both CCN and SM. The strains were used with the aim of determining their fermentation potential after being subjected to low-temperature storage (4°C) and carrier media. Before inoculation, the sorghum mixture was decontaminated using a water bath at 90°C for 30 min. After the heat treatment of the sorghum samples, pure strains were used. The strains were hydrated by incubating 1 g of starter culture in 9 mL of sterile 0.85% NaCl solution for 30 min and then mixed with sorghum slurry to an initial LAB population of 8 log (CFU/mL). The mixture was then fermented at 28°C, and the fermentation was monitored at intervals of 0, 12, 24, and 48 h. For comparison, a *ting* sample was prepared spontaneously, followed by incubation at 28°C for 48 h.

### 2.3. Determination of pH and Titratable Acidity

The pH was monitored using a pH meter (Hanna HI 8424) at intervals of 0, 12, 24, and 48 h. Total titratable acidity (TTA) was measured by titrating 0.1 N NaOH to a sample according to a method by GEA [14, 15]. Briefly, 10 g of fermented mixture was mixed with 100 mL of deionized water; the mixture was allowed to stand for 1 h while stirring gently. Then, 20 mL of the mixture was poured into a 100-mL Erlenmeyer flask, 0.5 mL of phenolphthalein added, and titrated with 0.1 N NaOH until a faint pink color persisted for 30 s [16]. Then, the percentage of lactic acid was calculated using formula [16]:

(1) $$\begin{array}{c} \text{Lactic acid }\left(\%\right)=\left(\frac{0.1\text{ M NaOH}\times \text{vol}.\text{of NaOH}\times 90.08}{\text{weight of the sample}}\right)\times 100. \end{array}$$

### 2.4. Determination of Total Soluble Solids (TSS) and Microbial Counts During Fermentation

The TSS (°Brix) were determined at 20°C using a refractometer [17]. Microbial counts were determined after serial dilutions, where 10 g of the fermented sorghum was mixed with 90 mL of 0.855% NaCl, followed by 10-fold serial dilutions. The spread plate method was performed. De Man–Rogosa–Sharpe (MRS) agar was used for LAB, while for spontaneous fermentation, MRS was still used as the standard for LAB enumeration based on previous studies that performed similar analyses [18–20]. Potato dextrose agar (PDA) supplemented with 1% chloramphenicol was used to enumerate yeasts and molds, while plate count agar (PCA) was used for total aerobic bacteria. Enterobacteriaceae were enumerated on violet red bile glucose agar (VRBGA). The MRS and VRBGA plates were incubated at 37°C for 24–48 h, PDA plates at 25°C for 48–96 h, and PCA plates at 30°C for 24–48 h. To ensure species specificity, the isolates were differentiated and counted based on morphological characteristics (colony color, shape, and size) throughout the microbial analysis. The isolates were further confirmed with presumptive analysis, including gram stain and catalase test.

### 2.5. Effect of Fermentation on the Functional Group of *Ting* Using Fourier Transform Infrared Spectroscopy (FTIR)

FTIR was used to analyze the functional groups of *ting* fermented with LAB preserved in SM and CCN (JASCO 4100, South Africa). Infrared spectrum data type was used with attenuated total reflectance (ATR) to identify the functional groups of the test compounds from fermented *ting*. The fermented *ting* samples were freeze-dried at −45°C for 48 h and analyzed in a dry state. A diamond crystal plate was used at a scan rate of 16 runs per scan, a resolution of 4 cm^−1^ in wavenumbers from 400 to 4000 cm^−1^. The FTIR device was equipped with a 1-mm diameter diamond ATR crystal, a 32× Reflachromat IR objective, and a sample stage. To subtract the effect of a previous run, a background spectrum was measured before running the test sample, and the surface of the ATR cell was cleaned with alcohol (ethanol) to ensure a clean background. The FTIR peaks were constructed using OriginPRO 2023 (graphing and analysis) software.

### 2.6. Sensory Evaluation of Sorghum Fermented With LAB

The use of humans for the consumer acceptability test was approved by the Faculty of Science, Ethics Committee, University of Johannesburg (2023-06-05/Zulu_Dlamini). Before evaluation, the panelists signed consent forms allowing authorization for the sensory evaluation. A total of 65 untrained panelists were selected based on their familiarity with fermented sorghum. The panelists were briefed about the product and how they should conduct the evaluation. Also, they were briefed on how to indicate the degree of likeness of the product on the questionnaire using a 9-point hedonic scale that ranged from 1 to 9, representing “extremely dislike” to “extremely like.” The coded samples were served in clean transparent PET tasting cups, and the panelists were given water to rinse their mouths in between tastings. The panelists were given instructions to taste and rate one sample at a time.

### 2.7. Statistical Analysis

The data was subjected to IMB SPSS Version 29.0 statistical analysis software to conduct a one-way analysis of variance (ANOVA). Differences in means were identified with Tukey's HSD multiple comparison test. All the parameters were analyzed in triplicates, and results were expressed as mean ± standard deviation (*p* < 0.05).

## 3. Results

### 3.1. Changes in pH and TTA During Fermentation of *Ting* With LAB Strains Preserved in SM and CCN Powder

**Figure 1:**
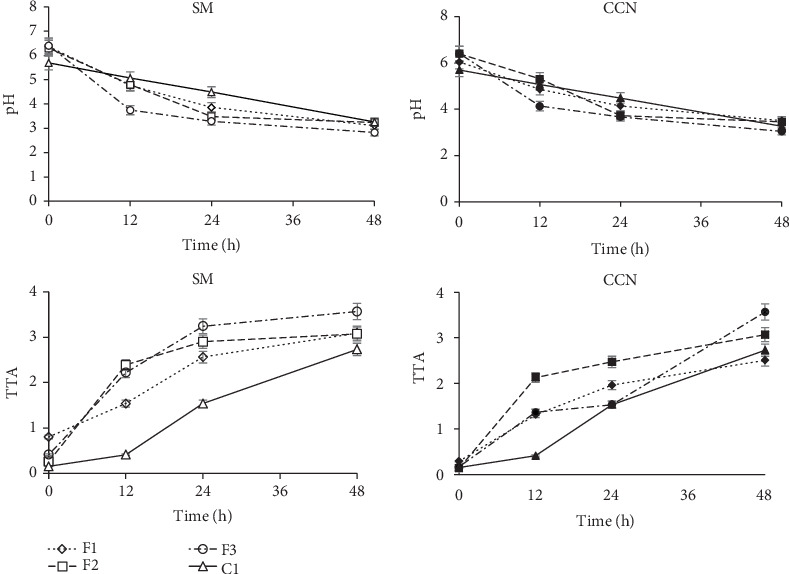
The pH and total titratable acidity of *ting* fermented using LAB prepared with skim milk (SM) and coconut (CCN) powder. F1—single *L. plantarum*, F2—single *L. brevis*, F3—mixed culture (F1 + F2), C—control (no starter culture). TTA: total titratable acidity.

The fermentation potential of single and mixed LAB strains that had been preserved in CCN and SM powder for 112 days was investigated in *ting.* Spontaneously fermented *ting* was used as the control. In this study, cryoprotection refers to the capacity of SM powder or CCN powder to sustain the viability and vitality of LAB cultures throughout the 112 days of storage before fermentation. To evaluate the effect of the cryoprotectant, changes in pH and TTA during the fermentation of ting were assessed. A significant (*p* < 0.05) reduction in pH was observed after 12 h in *ting* fermented with mixed strains (F3) when compared to the other treatments (Figure 1). This occurred with both starter cultures preserved in SM (6.41–3.75 pH reduction) and CCN powder (5.69–4.13 pH reduction) after 24 h. Overall, LAB strains preserved in SM had a more rapid reduction in pH and a lower final pH than LAB preserved in CCN. The viability and cell vitality were assessed before fermentation in our previous study, wherein SM supported a higher cell vitality and viability of *L. plantarum* compared to CCN [11]. Therefore, the rapid acidification observed here could be explained by the high vitality obtained in our previous study, which may have caused an increase in the acidification rates. Despite this, the pH of all the *ting* samples in this study was comparable as they fell within the pH range for fermented sorghum and *ting* (3.50–4.5) reported in the literature [17, 21, 22]. The pH of a fermented product serves as a measure of the concentration of hydrogen ions in a solution, which is largely influenced by the production of organic acids by LAB [23]. The rapid pH reduction in *ting* that was observed with the mixed strains could be because of the mixed strains' diverse metabolic capabilities, which allow the breakdown of diverse components within the fermentation substrate [14]. Also, mixed cultures can stimulate each other to achieve synergistic effects when compared to single cultures. In this study, the optimum pH (±3.5) for *ting* was reached within 24 h with single strains and within 12 h for mixed strains, while the spontaneously fermented *ting* took 48 h. The slow pH reduction in the spontaneously fermented *ting* could be due to its relatively low initial LAB count, which could have resulted in an extended lag phase. This finding confirms that the use of starter cultures, both single and mixed strains, reduces the fermentation time, with the mixed strains resulting in a more rapid acidification.

Titratable acidity determines the amount of free and bound hydrogen ions in a sample by titration with a base solution [15]. In general, a decrease in the pH is accompanied by an increase in titratable acidity, mainly because of the accumulation of organic acids. In this study, the highest increase in TTA occurred with the mixed strains after 12 h, and this was in line with pH reduction. Although mixed strains produced the highest TTA after 48 h (3.57%), this was not the case after 12 and 24 h, where single strains preserved with CCN powder produced more TTA than mixed strains. The exact reason for this is not known, but the available nutrients in CCN may have supported rapid metabolic activity for the production of certain acids during the initial step of fermentation. In general, a more rapid TTA production occurred with LAB preserved in SM powder when compared to CCN powder. A previous study reported the protective effect of milk proteins on *L. acidophilus*, which led to enhanced microbial stability and functional properties of this strain [24]. Therefore, the protective effect of milk proteins (casein, whey) and lactose may have supported LAB viability postpreservation, leading to a more rapid TTA production. Furthermore, the final TTA values for mixed strains and *L. brevis* were not significantly different from TTA prepared with SM versus CCN powder strains. This, in part, could be attributed to the heterofermentative nature of *L. brevis*, resulting in the production of lactic acid and other acids such as acetic acid [25]. Overall, the TTA at 48 h between groups (formulation) was not significantly different (*p* > 0.05) for all the formulations.

When compared to literature, the final TTA for mixed strains of *ting* from this study (3.57%) was higher than the reported range (0.57%–2.47%) for *ting* [17, 23]. The noticeable variation may be due to LAB strain differences. High TTA serves as one of the essential attributes required for the development of flavor, preservation, safety, and overall quality of fermented foods [26].

### 3.2. Change in TSS During Fermentation of *Ting* With LAB Strains Preserved With SM and CCN Powder

**Figure 2:**
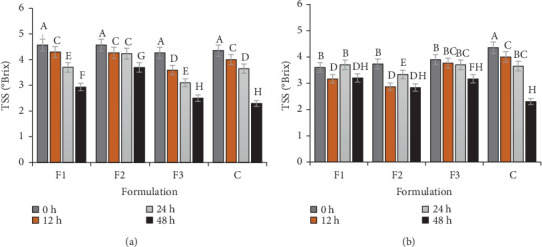
Total soluble solid (TSS) monitored during fermentation of sorghum using LAB prepared with (a) skim milk and (b) coconut powder. F1—single *L. plantarum*, F2—single *L. brevis*, F3—mixed culture (F1 + F2), C—control (no starter culture). Different alphabets (^ABCD^) indicate the significant difference (*p* < 0.05).

To further investigate the fermentation potential, changes in TSS during fermentation of *ting* prepared with single and mixed LAB strains preserved in SM and CCN were analyzed (Figure 2). The TSS on LAB prepared with SM significantly (*p* ≤ 0.05) decreased from 4.57 ± 0.31 to 2.93 ± 0.19 and 4.57–3.70 ± 0.10 after 48 h for *L. plantarum* (F1) and *L. brevis* (F2), respectively. The control sample had the lowest TSS (2.30 ± 0.06) after 48 h, and this was statistically similar to that of the mixed (F3) LAB (2.50 ± 0.21).

A constant decrease in TSS during fermentation was observed with *ting* fermented with all LAB preserved in SM, as well as on *ting* prepared with mixed strains in CCN, as shown in Figure 2. The steadier TSS decline with SM *ting* could possibly be due to its nutrient composition (milk proteins and lactose) that could have provided a more stable energy source or better protective effect for LAB during drying. In contrast, the TSS reduction on *ting* fermented with CCN LAB single strains showed fluctuations. The fluctuation could possibly be explained by the difference in vitality of the cultures since complex carbohydrates in the different substrates must first be digested into simple sugars before utilization, thus leading to different culture behavior. In addition, CCN could have influenced enzymatic activity within the LAB cells, leading to the production of amylases that hydrolyzed starch after 12 h of incubation [27]. A similar occurrence was reported by Ibrahim et al. [28], wherein fluctuations (3.3%, 6.1%, and 4.8% at 0, 12, and 28 h, respectively) in TSS during fermentation of sorghum dough with whey protein supplement were observed. These workers attributed the increase in TSS to solubilization of sorghum flour constituents because of fermentation. A decrease in TSS during fermentation is expected since it is associated with the metabolism of sugar by fermenting substrates [29].

The more stable TSS decline seen with mixed strains in both SM and CCN *ting* may be due to complementary metabolic activities between *L. plantarum* and *L. brevis*, resulting in a more efficient sugar breakdown [30]. Therefore, the use of mixed strains mitigated the TSS fluctuations observed in CCN *ting*, possibly through the diverse enzymatic actions of both strains leading to effective and consistent utilization of sugar substrates. The controlled fermentation with LAB ensured consistent sugar metabolism, unlike spontaneous fermentation, which, despite reaching the lowest TSS, showed unpredictable sugar utilization. The high variability in sugar breakdown patterns seen with spontaneous fermentation could possibly be due to the presence of non-LAB fermenters that may have contributed to erratic sugar utilization, further driving unpredictable TSS decline [31].

### 3.3. FTIR Analysis of *Ting* Fermented With LAB Strains

**Figure 3:**
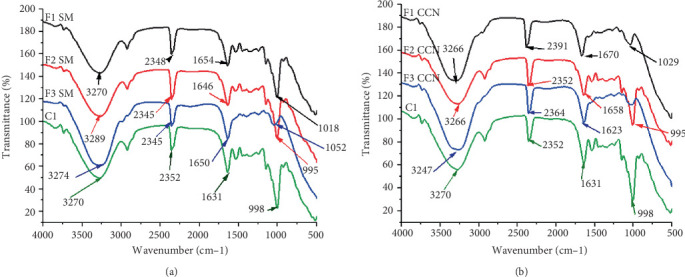
FTIR spectra of sorghum fermented for 48 h with LAB prepared with (a) skim milk powder and (b) coconut powder. F1—single *L. plantarum*, F2—single *L. brevis*, F3—mixed culture (F1 + F2), C—control.

Figure 3 shows the comparative FTIR spectra of *ting* fermented with single and mixed strains of *L. plantarum* and *L. brevis*. FTIR spectroscopy was used to determine the functional groups of *ting*. The spectrum of *ting* exhibited the presence of functional groups with distinct bands in four spectral regions, ranging between 3247–3289, 2348–2391, 1623–1670, and 995–1029 cm^−1^. The strong band at approx. 3300 1/cm corresponds to O–H stretching vibrations, and the ~2300–2800 cm^−1^ corresponds to C–H stretching vibration bands [32]. Notably, the detection of O–H stretching vibrations is associated with hydrogen bonding, which strongly suggests the presence of alcohols, phenols, and ethers when bonded [33]. The O–H stretching is associated with extensive hydrogen bonding, which may be indicative of fermentation-derived compounds like organic acids or polyphenols. These fermentation products are known to enhance the bioactive properties of fermented foods, and their formation during fermentation has been evidenced by FTIR in previous studies [34, 35]. In addition, these bonds may form water molecules if unbonded, further suggesting the potential presence of aromatic and flavor compounds [33].

The band at 1654 1/cm could be attributed to C=O, which is typically associated with amide I, while peaks at 1640 cm^−1^ likely correspond to conjugated carbonyl bonds that could be from flavonoids and esterified phytosterols. Such conjugated carbonyl absorptions could potentially arise from fermentation-induced transformations of phytochemicals. For example, the presence of flavonoids or other phenolic acids that contain C=O functional groups conjugated with aromatic systems [34]. This assignment is supported by literature showing carbonyl peaks around 1655–1645 cm^−1^ for flavonoid compounds (due to their aromatic carbonyl [C=O] coupled with C=C bonds) [36, 37]. The spectral shifts in the C=O region for the controlled fermentation (compared to uncontrolled) may thus indicate the formation or release of bioactive compounds due to microbial fermentation. The sharp peaks at 900 and 1030 cm^−1^ are potentially associated with phenolic compounds (flavonoids, tannins, glucosyl moieties), carbohydrates, ketones, or aldehydes containing the stretching vibrations of C-O-C and C-C or vibrational C-O-H bonds in carbohydrates and other polymeric structures [38, 39]. In addition, the spectral variations (995–1029 cm^−1^) indicate the occurrence of acid production and metabolic activity promoted by LAB-driven enzymatic breakdown of starches into fermentable sugars [39]. The spectral shift also shows that the efficiency of this breakdown was influenced by the differences in single versus mixed strain fermentations, leading to a possible impact on the final carbohydrate composition. Importantly, based on the spectral regions obtained in this study, we note that these assignments are suggestive rather than conclusive. However, they align with the known biochemical changes in fermented *ting*, such as increased phenolic content and the production of organic acids [35, 36].

In general, *ting* prepared with mixed LAB presented a wider spectrum and more pronounced shift suggesting stronger organic acid production which could enhance product flavor. It has been reported that when *L. plantarum* is in combination with other LAB strains, it releases bound polyphenols and flavonoids from plant matrices [32, 40]. This was reflected in stronger O–H and C=C spectral signals. The single LAB strains caused more fluctuations in functional group intensities potentially due to pronounced variability in organic acid formation. Some LAB, including *L. brevis*, have weaker phenolic-degrading enzymes which delay the production of bioactive flavonoids and phenolic acids leading to lower phenolic metabolism [38, 41].

The spectra for *ting* prepared with SM and CCN LAB cultures increased compared to the control sample, suggesting enhanced biochemical activity due to LAB metabolism. The stronger peak intensities with SM *ting* when compared to CCN *ting* suggest enhanced metabolic activity, potentially leading to higher flavonoid conversion or protein interactions and improved bioavailability of amino acids, which play a role in flavor development and nutritional quality.

*Ting* was previously reported to contain a high concentration of flavanol glycosides and potential protein buildup in the form of amino acids, which could account for the broader peak's bands in this part of the spectrum [36]. On the contrary, for *ting* prepared with LAB preserved in CCN, the regions 3266 and 1623 showed a decrease in intensity. These changes may be associated with the reduction in hydroxyl groups (O–H) and carbonyl-containing compounds (acetaldehyde, formaldehyde, diacetyl (2,3-butanedione), acetone) caused by the conversion of carbonyl compounds from LAB. Moreover, the region around 3266 represents N–H stretch, which indicates the availability of amines, amides, and protein molecules. In agreement with the previously reported outcome [42], wherein a reduction in intensity was observed from these regions around 1700 cm^−1^ for *ting* samples. The band intensity of SM increased more than that of CCN; this shift could be due to the interaction between OH groups of cryoprotectants and membrane phospholipids of starter cultures in *ting* [42].

### 3.4. Changes in Microbial Quality During Fermentation of *Ting*

The microbial quality of *ting* during fermentation was monitored through observing changes in TAC, LAB, and yeast and molds. With TAC, there were no detected counts after 12 h (Table 2). Thereafter, TAC counts were detected after 24 h, with the highest count (3.74 log CFU/mL) occurring on both F3 SM (mixed strains) and F1 CCN (*L. b*). However, between 24 and 48 h of fermentation, the TAC increase was not more than 1 log CFU/mL for all the formulations, and this may be regarded as not significant. In general, high TAC is expected in fermented foods because of the normal microbial flora present [43]. Despite this, the TAC in food of good quality should not exceed 5 × 10^5^ CFU/g. Thus, the low TAC counts here compared to the general microbial requirement suggest that the *ting* was of good quality and could potentially have an acceptable shelf-life [44].

As expected, the extension of the fermentation period increased the LAB counts. A rapid increase in LAB counts was observed after 24 h, with the mixed LAB strains (F3) preserved in CCN powder showing the highest LAB count increase (3.96 log CFU/mL) when compared to 0 h counts. When the LAB count in single strains was compared with the mixed strains, the single strains showed lower counts. Among single strains, *L. plantarum* counts were significantly higher than *L. brevis* counts. Overall, the highest increase in LAB counts was observed with the mixed LAB on both SM (3.38 log CFU/mL increase) and CCN powder (4.87 log CFU/mL). As expected, the least increase in LAB counts (2.02 log CFU/mL) occurred with the control (spontaneous fermentation), while all the controlled fermentation had the highest LAB increase. The increase noted with LAB counts is an indication of microbial activity during fermentation. There were no significant differences in TAC counts between the mixed strain of SM and CCN *ting*. However, significant differences were noted for single strains, with CCN preserved strains showing higher counts when compared to SM strains. Importantly, an increase in TTA was observed in this study. Such increase could have been due to the continued LAB activity, while the observed decrease in TSS was attributed to the ability of LAB strains to convert sugars into acids and other fermentation by-products. As expected, the increase in LAB counts corresponded with a decrease in pH.

For all formulations, the total yeast and mold count and ENBs were not detected. The absence of yeasts and molds suggests the application of effective hygiene and sanitation practices, while ENBs are not only reliable hygiene indicators but may also indicate fecal contamination of food products [44]. Based on GCC Standard Organization (GSO) [45] and FAO [46] ENB, yeasts and molds should not exceed 2 and 3 log CFU/g, respectively, in cereal food. Therefore, the results show that the formulations prepared in this study could be shelf stable and may be deemed safe for human consumption when compared to the microbial criteria of GSO [45]. Other studies have also reported ENB counts of less than 10 CFU/g on fermented *ting* [18].

### 3.5. Consumer Acceptability of *Ting* Fermented With LAB Preserved With CCN and SM Powder as Carrier Media

The mixed LAB culture formulation was used to prepare *ting* samples for sensory evaluation due to its superior performance in terms of microbial quality and acidification kinetics during fermentation. The sensory profile of fermented *ting* prepared with mixed strains of LAB is summarized in Table 3. In terms of appearance, the control sample was the most preferred with the highest score (7.68 ± 1.46), followed by SM (6.78 ± 1.53), while *ting* prepared with mixed LAB preserved in CCN was the least preferred sample (6.44 ± 1.14). Interestingly, *ting* from CCN mixed strains showed the highest flavor score (6.52 ± 1.47), while that prepared with SM mixed strains and control *ting* had statistically similar (*p* ≤ 0.05) flavor scores. This was despite the fact that the pH and TTA for *ting* from both mixed strains were statistically similar. There was no significant difference between the aroma score for the controlled (CCN *ting*) versus spontaneous fermentation, while SM *ting* was the least preferred. With mouthfeel, *ting* produced with the CCN preserved strain had the highest score (6.56 ± 1.72) followed by the spontaneous fermented *ting* (6.30 ± 1.07) which was not significantly different from that of SM strains (5.69 ± 1.41).

Overall, there was a significant difference between the consumer acceptability for *ting* prepared with SM strains and *ting* prepared with CCN strains. *Ting* prepared with CCN preserved mixed strains appeared to be the most preferred sample due to its highest overall score (6.95 ± 1.86), followed by the SM *ting* (5.67 ± 1.52) which was statistically similar to the control. It has been reported that the taste and flavor of food products greatly influence the acceptability of the product [19]. Therefore, the high flavor score for CCN *ting* could explain its overall acceptability.

### 3.6. Principal Component Analysis (PCA) of Variables Analyzed for Fermented *Ting*

**Figure 4:**
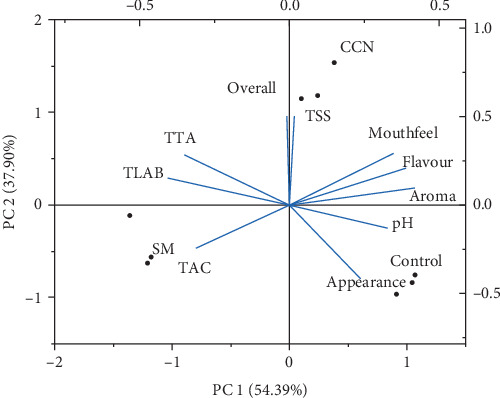
Biplot for principal component analysis (PCA) of *ting* fermented with LAB from coconut and skim milk powder. Potential of hydrogen (pH), total titratable acidity (TTA), total soluble solids (TSS), total lactic acid bacteria (TLAB), and total aerobic count (TAC).

A biplot from the PCA was compiled and presented to streamline the interpretation of the data. This was done to explore the interrelationships between physicochemical (TSS, TTA, pH), microbial, proximate, and sensory parameters of the mixed strain formulation. As illustrated in Figure 4, the 16 measured variables of *ting* samples were reduced to two interpretable principal components (PC1 and PC2) that described about 92.29% of the total variation in the samples, with 54.39% for PC1 and 37.90% for PC2. Figure 4 shows that variables that mainly contributed positively to the PC1 axis were flavor, aroma, TSS, pH, appearance, and mouthfeel, while TAC described the negative side of PC1. The TTA exhibited a positive correlation with TLAB, indicating that as the total LAB count increased, so did the titratable acidity. This correlation is attributed to the presence of organic acids, particularly lactic acid, which is the primary organic acid produced by LAB during fermentation. Overall score, TLAB, and TTA were positively correlated with the PC2 axis, with TCA describing the negative side of the same axis. According to Figure 4, there was a heterogeneity between CCN, SM, and the control sample, with CCN and control separating positively to the right, while the SM separated to the negative side left of PC1. This means that the *ting* prepared with CCN powder (controlled fermentation) had the best overall mouthfeel, flavor, and aroma, while for the control sample (spontaneous fermentation) this means that the sample was more accepted in terms of its appearance.

### 3.7. Conclusion

In this study, two distinct LAB strains (*L. plantarum* and *L. brevis*) previously preserved in CCN and SM powder were comparatively analyzed for changes in physicochemical properties, microbial quality, sensory profile, and functional groups during the fermentation of *ting*. The LAB strains preserved in SM and CCN resulted in *ting* with a more rapid reduction in pH, higher TTA, and lower final pH than the naturally fermented sample. Overall, the TSS of *ting* was influenced by LAB starter cultures, with SM preserved mixed strains performing better, with a constant decrease in TSS compared to mixed strains in CCN. Significant differences were noted for TAC counts in single strains, where *ting* produced with CCN preserved strains showed higher counts compared to SM strains. The preservation carrier media influenced the increase in LAB, with a significantly higher LAB count noted in *ting* fermented with mixed strains for both SM and CCN compared to the control. Furthermore, the *ting* prepared with single strains in CCN resulted in higher LAB counts compared to that of SM strains. With all the formulations, the total yeast and mold count and ENBs were not detected. Additionally, FTIR analysis confirmed the presence of similar functional groups for SM and CCN strain samples. With consumer acceptability, *ting* prepared with CCN strain appeared to be the most preferred sample due to its highest overall score, followed by the SM sample. Overall, the findings demonstrate that the use of CCN to preserve LAB strains can potentially contribute to the stability and quality fermentation of traditional foods. However, the current paper suggests that further research must focus on the identification of volatile compounds and organic acids that could influence the flavor profile of *ting*. Also, due to the fluctuations in TSS observed, future studies should explore enzyme activity assays to determine the role of carbohydrates in TSS fluctuations.

## Figures and Tables

**Table 1:** Formulations for *ting* samples prepared with both CCN and SM cultures.

**Ingredients**	**F1 (*L. p*)**		**F2 (*L. b*)**		**F3 (*L. p + L. b*)**		**Control**	
	**g**	**%**	**g**	**%**	**g**	**%**	**g**	**%**
Sorghum	50.00	24.69	50.00	24.69	50.00	24.69	50.00	25.00
LAB culture	2.50	1.23	2.50	1.23	2.50	1.23	0.00	—
Water	150.00	74.07	150.00	74.07	150	74.07	150.00	75.00
Total	202.50	100	202.50	100	202.50	100	200.00	100.00

*Note:* Formulation 1 (F1)—single *L. plantarum* (*L. p*), Formulation 2 (F2)—single *L. brevis* (*L. b*), and Formulation 3 (F3)—mixed culture (*L. p + L. b*) and control (spontaneously fermented).

**Table 2:** Microbial quality of *ting* fermented with LAB strains preserved in SM and CCN powder (log CFU/mL).

**Item**	**Time (h)**	**Formulation**						
		**F1 SM**	**F2 SM**	**F3 SM**	**F1 CCN**	**F2 CCN**	**F3 CCN**	**Control**
TAC	0	ND	ND	ND	ND	ND	ND	ND
	12	ND	ND	ND	ND	ND	ND	ND
	24	3.41^Ab^ ± 0.03	3.09^Bc^ ± 0.08	3.74^Aa^ ± 0.00	3.53^Aa^ ± 0.01	3.74^Aa^ ± 0.01	3.63^Aa^ ± 0.01	3.39^Ab^ ± 0.08
	48	3.47^Ab^ ± 0.02	3.51^Ab^ ± 0.02	3.76^Aa^ ± 0.00	3.76^Aa^ ± 0.00	3.81^Aa^ ± 0.01	3.72^Aa^ ± 0.02	3.47^Ab^ ± 0.02

LAB	0	5.83^Dd^ ± 0.02	5.95^Cb^ ± 0.02	6.50^Da^ ± 0.05	5.90^Cc^ ± 0.02	5.77^Bd^ ± 0.02	5.21^De^ ± 0.03	4.90^Cf^ ± 0.01
	12	6.93^Cc^ ± 0.02	6.80^Bd^ ± 0.02	7.74^Cb^ ± 0.02	5.73^Cf^ ± 0.01	5.93^Be^ ± 0.03	7.88^Ca^ ± 0.01	5.74^Bb^ ± 0.05
	24	8.89^Bc^ ± 0.04	7.97^Bf^ ± 0.12	9.06^Bb^ ± 0.04	8.13^Be^ ± 0.15	9.03^Ab^ ± 0.13	9.17^Ba^ ± 0.05	8.64^Ad^ ± 0.04
	48	9.73^Ac^ ± 0.23	8.84^Ad^ ± 0.02	10.88^Aa^ ± 0.03	10.00^Ab^ ± 0.01	9.01^Ad^ ± 0.02	10.06^Ab^ ± 0.06	8.92^Ad^ ± 0.09

YM	0	ND	ND	ND	ND	ND	ND	ND
	12	ND	ND	ND	ND	ND	ND	ND
	24	ND	ND	ND	ND	ND	ND	ND
	48	ND	ND	ND	ND	ND	ND	ND

ENB	0	ND	ND	ND	ND	ND	ND	ND
	12	ND	ND	ND	ND	ND	ND	ND
	24	ND	ND	ND	ND	ND	ND	ND
	48	ND	ND	ND	ND	ND	ND	ND

*Note:* F1—single *L. plantarum*, F2—single *L. brevis*, and F3—mixed culture (F1 + F2) and control (no starter culture). The values with similar *small letter* alphabets in each row (between formulation) indicate no significant difference (*p* ≥ 0.05), while the values with different *capital letter* alphabets in each column (per incubation time (hour)) indicate a significant difference. TAC and LAB were statistically analyzed separately.

Abbreviations: ENT, Enterobacteriaceae; LAB, lactic acid bacteria count; ND, not detected; TAC, total aerobic count; YM, yeast and mold count.

**Table 3:** Consumer acceptability evaluation of *ting* fermented with LAB prepared with skim milk (F3 SM) and coconut (F3 CCN) powder.

**Sample ID**	**Appearance**	**Flavor**	**Aroma**	**Mouthfeel**	**Overall**
Control	7.68 ± 1.46^a^	6.42 ± 1.55^a^	6.87 ± 1.52^a^	6.30 ± 1.07^b^	5.30 ± 1.56^a^
F3 SM	6.78 ± 1.53^b^	5.64 ± 1.74^b^	6.07 ± 1.43^b^	5.69 ± 1.41^b^	5.67 ± 1.52^a^
F3 CCN	6.44 ± 1.14^c^	6.52 ± 1.47^a^	6.75 ± 1.58^a^	6.56 ± 1.72^a^	6.95 ± 1.86^a^

*Note:* Values with similar alphabets in the column indicate no significant difference (*p* ≥ 0.05). F3—mixed culture and control (no starter culture). CCN—*ting* prepared with strains preserved in coconut. SM—*ting* prepared with strains preserved in skim milk. Values with different alphabets in each column indicate significant differences (*p* < 0.05).

## Data Availability

The data that support the findings of this study are available on request from the corresponding author. The data are not publicly available due to privacy or ethical restrictions.

## General Statement

*Practical Application*. This article will give insight into the use of alternative carrier media for starter culture development to cater for traditionally fermented foods.
